# Radiographic outcomes of femur fractures following SIGN Fin nailing in low- and middle-income countries

**DOI:** 10.1097/OI9.0000000000000141

**Published:** 2021-07-29

**Authors:** Zachary H. Birner, Scott J. Hetzel, Nathaniel M. Wilson, Paul S. Whiting

**Affiliations:** Department of Orthopedics and Rehabilitation, University of Wisconsin School of Medicine and Public Health, Madison, WI

**Keywords:** alignment, femur fracture, intramedullary nail, resource-limited setting, SIGN

## Abstract

**Objective:**

To measure the effectiveness of the Surgical Implant Generation Network (SIGN) Fin nail for achieving satisfactory postoperative radiographic alignment following femoral shaft fractures.

**Methods:**

Femoral shaft fractures stabilized with the SIGN Fin nail were identified using the SIGN Online Surgical Database. A random number generator was used to identify 500 femur fractures fixed within 6 weeks of injury for which postoperative radiographs were available. Fractures were classified using OTA/AO and Winquist-Hansen classification systems. Deviation from anatomic alignment was measured on anterior-posterior and lateral radiographs using an on-screen protractor tool. Other clinical variables recorded in the SIGN Online Surgical Database were also analyzed. Simple logistic regression was used to assess for associations between subject and surgical characteristics and misalignment status. Intra- and inter-rater agreement was assessed with intraclass correlation coefficient (ICC).

**Results:**

The overall rate of malalignment >5° was 9.4%. Factors associated with increased incidence of malalignment include older age, increased time to surgery, distal diaphyseal location, closed (vs open) reduction, degree of comminution, and fracture classification. Intra-rater ICC was 0.70 (0.52, 0.82) in the coronal plane and 0.55 (0.32, 0.72) in the sagittal plane. Inter-rater ICC was 0.37 (0.08, 0.60) and 0.32 (0.05, 0.54), respectively.

**Conclusion:**

The SIGN Fin nail is an effective implant for fixation of femoral shaft fractures in resource-limited regions, achieving rates of satisfactory postoperative alignment comparable to that of the standard SIGN nail as well as femoral shaft fractures treated in North American Trauma Centers. Further research is required to investigate rotational alignment and long-term clinical outcomes for the SIGN Fin nail.

**Level of evidence:**

IV.

## 1 Introduction

Roughly 1.35 million people die in motor vehicle collisions globally each year with an additional 20 to 50 million suffering nonfatal injuries, many of which contribute to lasting disability.^[1]^ According to the World Health Organization, in 2016 road traffic injury became the fifth leading cause of disability adjusted life years lost, up from tenth place in 2000.^[2]^ While the World Health Organization's global status report on road safety revealed that positive measures have been taken, such measures are far more prevalent in high- and middle-income countries compared with low-income countries.^[3]^ This has led to decreased rates of injury in high-income countries and a simultaneous rise in injury rates among low- and middle- income countries.^[4]^

**Figure 1:**
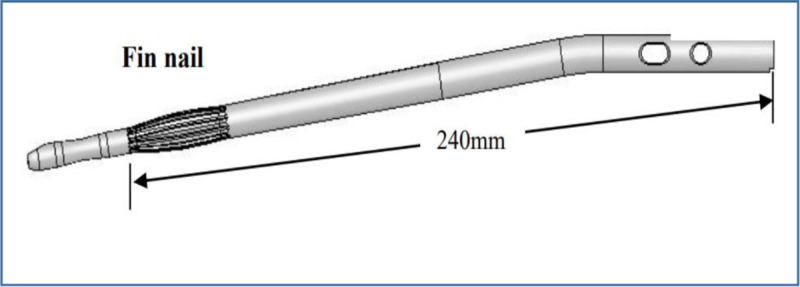
Graphic representation of a SIGN Fin nail.

The Surgical Implant Generation Network (SIGN) is a nonprofit organization with a mission of diminishing the global disparity in fracture care. The SIGN intramedullary nail system was developed as an effective and affordable option for treatment of femoral, tibial, and humeral fractures in resource-limited settings, where power instrumentation, fluoroscopy, and specialized fracture tables are not always available. The SIGN Fin nail (Fig. 1) differs from the standard SIGN nail in that it does not require interlocking screw placement in the leading end of the nail. Instead, the leading end of the nail has fins, which achieve an interference fit within the intramedullary canal.^[5]^ This design modification further simplifies intramedullary fixation of long-bone fractures, particularly in the absence of fluoroscopy.

To date, several studies have been published assessing the efficacy of the SIGN intramedullary nailing system for fixation of femoral shaft fractures. Carsen et al^[6]^ performed a retrospective review of more than 500 femur fractures stabilized with a standard SIGN nail and concluded that the incidence of malalignment was comparable to that of North American trauma centers. In a case-control study at 2 African hospitals, Wilson et al^[7]^ reported no difference in average deviation from anatomic alignment (DFAA) between femur fractures stabilized with the SIGN Fin nail and the standard SIGN nail. In a separate study, the same investigators reported satisfactory alignment in a cohort of more than of 250 femur fractures stabilized with the SIGN Fin nail with minimum 6 month follow-up.^[8]^

While the 2 aforementioned studies investigating the SIGN Fin nail have shown promising results for satisfactory postoperative alignment, neither study represented a cross-section of global SIGN sites. As such, the generalizability of these results is limited. The current study, therefore, aims to measure the effectiveness of the SIGN Fin nail for achieving satisfactory postoperative radiographic alignment following femoral shaft fracture fixation. Our study design is a retrospective analysis of prospectively collected data entered into the SIGN Online Surgical Database (SOSD) similar to that performed by Carsen et al,^[6]^ but specific to the SIGN Fin nail rather than the standard SIGN nail.

## 2 Methods

### 2.1 Study design and patient selection

Our study was deemed to be exempt by our Institutional Review Board. We performed a retrospective analysis of the SOSD, a deidentified multicenter database managed by SIGN Fracture Care International. We performed a query of the entire SOSD using the following inclusion criteria: all closed femur fractures treated in World Bank-defined low- and middle-income countries using a SIGN Fin nail. Cases meeting these criteria were exported into a spreadsheet (Microsoft Excel, Microsoft Inc, Redmond, Washington) and sorted by Case ID into a random order. Using the SOSD, cases in the spreadsheet were reviewed in sequential order to ensure that the inclusion/exclusion criteria were met. Exclusion included lack of postoperative x-rays, poor x-ray quality, pathologic fractures, fractures involving the femoral metaphysis (as defined by a distance from the proximal or distal articular surface less than the width of the respective proximal or distal metaphysis), surgery performed for subacute fractures (>6 weeks following injury) or nonunion, and the use of any adjunctive implants for fixation (i.e., unicortical plates, lag screws, blocking screws, cerclage wires, etc). Cases were reviewed until a total of 500 cases were included in the final analysis.

### 2.2 Fracture alignment measurements and classification

**Figure 2:**
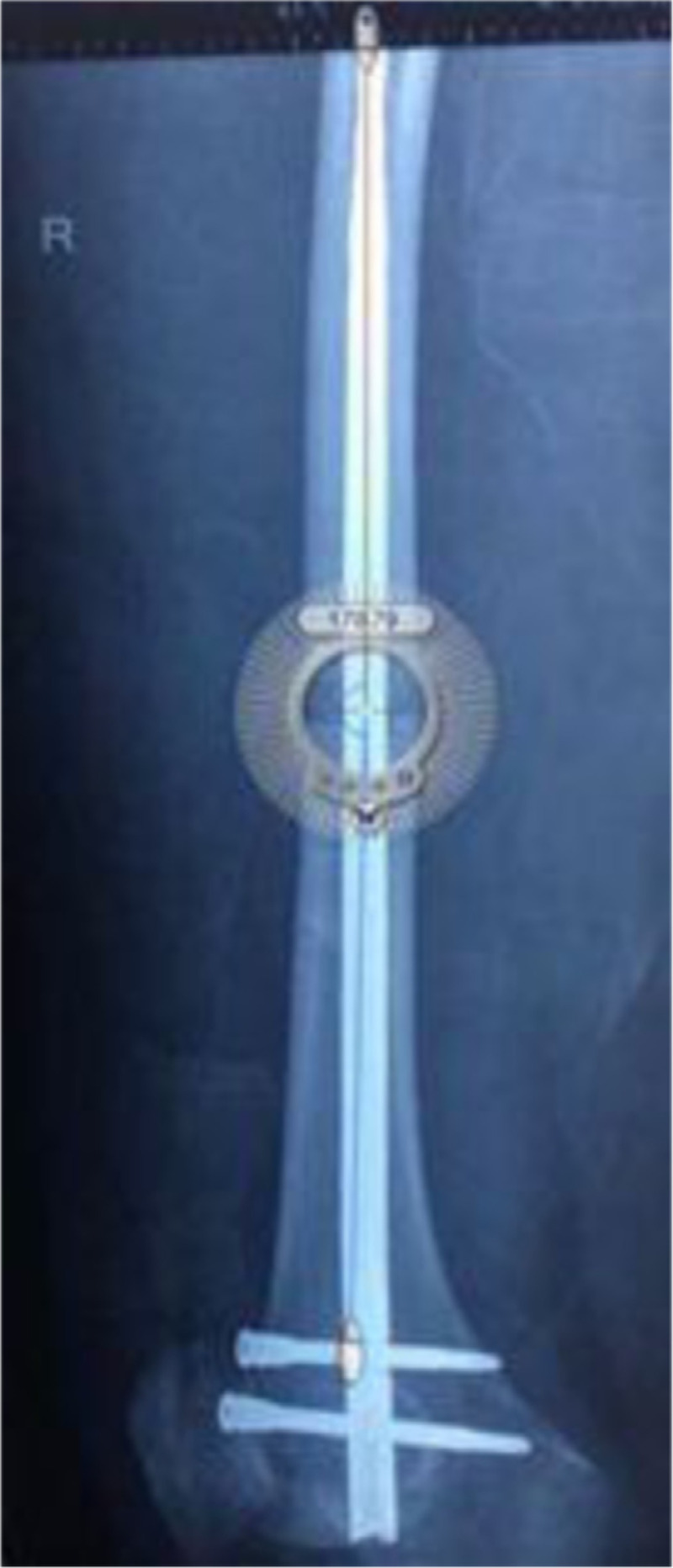
Screen protractor tool.

Fractures were analyzed in both the coronal and sagittal planes using anterior-posterior and lateral radiographs, respectively. Fracture alignment was measuring using degrees of DFAA, and measurements were made utilizing an on screen protractor tool (Screen Protractor; Iconico Inc, New York, New York) as previously described.^[7,8]^ which was layered over films from the database (Fig. 2). Absolute values for DFAA were recorded along with the direction of the deformity (varus or valgus on the anterior-posterior view, and procurvatum or recurvatum on the lateral view). For categorical analysis, malalignment was defined as greater than 5° of DFAA as previously described.^[7,8]^ Fractures were classified using the OTA/AO and Winquist Hansen classification systems for diaphyseal femur fractures. Furthermore, clinical outcome variables available in the database were also analyzed including patient age, sex, time delay to surgery (acute defined as < 4 weeks, subacute defined as 4 weeks-6 months), fracture location (distal, middle, or proximal third of the diaphysis), and surgical reduction method (open vs closed). Alignment measurements and fracture classifications were determined by a trained observer under the direction of the senior author. When ambiguity in fracture classification was encountered, classification determinations were resolved by the senior author. Fifty cases were then randomly selected for repeat measurements by the trained observer and measurements by the senior author to calculate intra- and inter-rater reliability measurements using the intraclass correlation coefficient (ICC).

### 2.3 Statistical analysis

Cohort characteristics were summarized using N (%) or mean (SD) based on the statistical distribution of the particular characteristic. Malalignment was determined as > 5° of DFAA in either AP or lateral planes. Simple logistic regression was used to assess for associations between subject and surgical characteristics and misalignment status. Inter-rater agreement was assessed with ICC (2,1) and intra-rater agreement was assessed with ICC(3,1).^[9]^

## 3 Results

The original query of the SOSD yielded 6865 cases from 215 unique institutions, which were then sorted in a random order for radiographic analysis as described above. During analysis, a total of 710 fractures were evaluated, and 210 cases were excluded for the following reasons: 13 cases had no postoperative x-rays in the database; 25 cases had x-rays of insufficient quality for alignment measurement; 34 cases represented treatment of a pathologic fracture, subacute fracture (>6 weeks from injury) or nonunion; 23 cases were too proximal (peritrochanteric/subtrochanteric fractures); 73 cases were too distal (metaphyseal supracondylar fractures); 22 cases employed supplemental fixation; and 20 cases were stabilized with standard SIGN nails but mislabeled as SIGN Fin nails.

A summary of the demographics for the 500 cases (from 105 unique institutions) meeting inclusion and exclusion criteria is outlined in Table 1. The average age of patients was 32.1 years, with 75.6% of the final cohort being male. 89.8% of surgical procedures (449 of 500) were performed via a retrograde approach. Fractures were most often located in the mid diaphysis (71.4% of all fractures) and categorized as OTA/AO type 32A fractures (transverse or short-oblique) with minimal to no comminution (Winquist Hansen Grade 0 or 1, see Table 1).

**Table 1:** Summary of study cohort.

Variable	Total N = 500
Age—years	32.1 (12.9)
Gender—male	378 (75.6%)
Surgical timing—subacute	55 (11.2%)
Approach—retrograde	449 (89.8%)
OTA/AO class	
32A	366 (73.2%)
32B	117 (23.4%)
32C	17 (3.4%)
Winquist-Hansen	
0	175 (35.0%)
1	156 (31.2%)
2	80 (16.0%)
3	73 (14.6%)
4	16 (3.2%)
Location	
Middle	357 (71.4%)
Distal	122 (24.4%)
Proximal	21 (4.2%)
Fracture reduction-closed	80 (16.0%)
Varus status (n = 465)	283 (60.9%)
Recurvatum status (n = 248)	120 (48.4%)
reported as mean (SD), N (%)	

Overall postoperative malalignment >5° in either the coronal or sagittal plane was identified in 47 of 500 cases (9.4%), most commonly in varus and/or recurvatum. As shown in Table 2, multiple patient and surgical factors were associated with increased incidence of malalignment. Older age was associated with an increased risk of malalignment; patients with well aligned fractures had an average age of 31.7 years compared with an average age of 36.3 years in malaligned fractures (odds ratio per year of 1.02, 95% CI 1.00–1.05). Time from injury to surgery of >4 weeks resulted in greater risk of malalignment (18.2% vs 8.1%, *P* = .018), representing 2.53 times increased odds of malalignment (95% CI 1.18–5.46). Fractures located in the distal diaphysis were more likely to be malaligned compared with those in the mid diaphysis (23.0% vs 4.5%, *P* < .01) representing an odds ratio of 6.35 (95% CI 3.30–12.22) for malalignment in distal diaphyseal fractures. While fractures of the proximal diaphysis also had a higher rate of malalignment (14.3%), this trend did not reach statistical significance (*P* = .06).

**Table 2:** Association of patient and surgical characteristics with malalignment >5° in either plane.

Variable	Well aligned	Malaligned	OR (95% CI)	*P* value
Age-yr	31.7 (12.6)	36.3 (14.8)	1.02 (1.00–1.05)	.020

	Total N	Malaligned	OR (95% CI)	*P* value
Gender				
F	122	9 (7.4%)	Reference	–
M	378	38 (10.1%)	1.40 (0.66–2.99)	.380
Surgical timing				
Acute	434	35 (8.1%)	Reference	–
Subacute	55	10 (18.2%)	2.53 (1.18–5.46)	.018
OTA/AO class				
32A	366	25 (6.8%)	Reference	–
32B	117	13 (11.1%)	1.70 (0.84–3.45)	.138
32C	17	9 (52.9%)	15.34 (5.45–43.21)	<.001
Winquist Hansen				
0	175	10 (5.7%)	Reference	–
1	156	12 (7.7%)		
2	80	5 (6.2%)	2.44 (1.33–4.47)	.004
3	73	12 (16.4%)		
4	16	8 (50.0%)		
Location				
Middle	357	16 (4.5%)	Reference	–
Distal	122	28 (23.0%)	6.35 (3.30–12.22)	<.001
Proximal	21	3 (14.3%)	3.55 (0.95–13.31)	.060
Fracture reduction				
Open	420	29 (6.9%)	Reference	–
Closed	80	18 (22.5%)	3.91 (2.05–7.47)	<.001
Varus/valgus (n = 465)				
Valgus	182	12 (6.6%)	Reference	–
Varus	283	35 (12.4%)	3.43 (1.29–9.13)	.014
Procurvatum/recurvatum (n = 248)				
Procurvatum	128	11 (8.6%)	Reference	–
Recurvatum	120	20 (16.7%)	2.48 (1.03–5.97)	.044

Open reduction was associated with decreased incidence of malalignment (6.9% vs 22.5%, *P* < .01) compared with closed reduction. This resulted in an odds ratio for malalignment of 3.91 (95% CI 2.05–7.47) with closed reduction vs open reduction. When fractures were grouped by Winquist Hansen classification, fractures classified as types 2, 3, or 4 were more likely to be malaligned than those classified as types 0 and 1 (odds ratio of 2.44 (95% CI 1.33–4.47), *P* = .004). Similarly, fractures classified as OTA/AO 32C were most likely to be malaligned, with an odds ratio for malalignment of 15.34 (95% CI 5.45–43.21, *P* < .001) compared with 32A fractures.

Intra-rater and inter-rater reliability was calculated for both the coronal and sagittal plane measurements. Intra-rater ICC was 0.70 (0.52, 0.82) in the coronal plane and 0.55 (0.32, 0.72) in the sagittal plane. Inter-rater ICC was 0.37 (0.08, 0.60) and 0.32 (0.05, 0.54) in the coronal and sagittal planes, respectively. Of the 50 cases selected for intra- and inter-rater reliability measurements, 7 fractures (14%) were categorized as malaligned on the initial measurements (14%), and the average DFAA for all 50 cases was 2.2° in the coronal plane and 3.0° in the sagittal plane. Repeat measurements by the same observer categorized 5 fractures (10%) as malaligned with an average DFAA for all 50 cases of 1.7° in the coronal plane and 2.8° in the sagittal plane. Measurements performed by the senior author categorized 3 fractures (6%) malaligned with average DFAA measurements of 1.3° in the coronal plane and 2.0° in the sagittal plane.

## 4 Discussion

Rates of road traffic injury continue to rise in developing nations, and the resulting injuries represent a leading cause of disability adjusted life years lost.^[1]^ Barriers to health care access and inadequate physical and human resources likely account for a significant portion of the disparity in outcomes observed between high-income countries and low- and middle-income countries. The SIGN intramedullary nailing system has been developed to address this disparity by promoting equality in fracture care worldwide. The purpose of our study was to evaluate immediate postoperative alignment in a large cohort of femur fractures stabilized with the SIGN Fin nail in low- and middle-income countries.

We observed an overall rate of malalignment >5° of 9.4%, results which are comparable to those previously reported for the standard SIGN nail (10%)^[6]^ as well as malalignment rates previously reported for a large cohort of patients treated at a high-volume North American trauma center (9%).^[10]^ To our knowledge, the current study represents the largest cohort of femur fractures stabilized with the SIGN Fin nail. Wilson et al^[7]^ previously published a case-control study comparing outcomes of femoral shaft fractures stabilized with the SIGN Fin nail vs a standard SIGN nail. The authors did not observe any cases of malalignment >5° among the 28 fractures stabilized with the SIGN Fin nail. The same authors recently published a case series of 249 fractures fixed with the SIGN Fin nail with minimum 6-month follow-up and reported a rate of malalignment (defined in that study as DFAA >10° in any plane) of 6%.^[8]^ Liu et al^[11]^, in a study comparing the SIGN Fin nail to the standard SIGN nail, documented no significant difference in rate of reoperation, infection, limb length discrepancy, nonunion, or angular malalignment at 1 year of follow-up. The current study adds to this growing body of literature supporting the utility of the SIGN Fin nail for achieving satisfactory postoperative alignment of femoral shaft fractures in low- and middle-income countries.

Our study also identified a number of patient and surgical variables associated with malalignment. Fracture comminution, distal diaphyseal fracture location, and time from injury to surgery >4 weeks were all associated with increased risk of malalignment in our study, findings consistent with those reported by Carsen et al^[6]^ for femur fractures stabilized using the standard SIGN nail. While those authors also reported significantly increased rates of malalignment for proximal diaphyseal fractures as well, the trend we observed for greater odds of malalignment among proximal fractures did not reach statistical significance (*P* = .06). This can likely be attributed to the relatively small proportion of proximal diaphyseal fractures in our study (24 of 500 cases, a mere 4.8% of the entire study cohort).

We also identified increasing age as a risk factor for malalignment, with an odds ratio of 1.02 (95% CI 1.00–1.05) per year of age. Age-related decreases in bone mineral density are well documented in the literature,^[12]^ but since bone mineral density was not measured in our study cohort, we cannot definitively attribute these observed differences to bone quality alone. Our study also identified significantly increased odds of malalignment with closed reduction compared with open reduction (OR 3.91, 95% CI 2.05–7.47). Open reduction obviously offers the benefit of direct fracture visualization to aid in fracture alignment, and this approach is utilized frequently in settings which lack fluoroscopy. The benefits of open reduction, however, must always be weighed against the risks of potential complications, such as surgical site infection.^[13]^

Strengths of our study include several components of the methodology, particularly the random sampling of SIGN Fin nail cases from the SOSD. This feature of the study design was intended to capture an accurate cross-section of institutions performing femur fracture care in low- and middle-income countries. By analyzing alignment on immediate postoperative radiographs, we have eliminated the potential bias associated with differential follow-up rates between institutions and regions. As such, our study represents a pragmatic assessment of the performance of the SIGN Fin nail in femoral shaft fracture stabilization. Prior studies^[8,11]^ have already demonstrated the SIGN Fin nail is satisfactory and comparable to the standard SIGN nail at up to 1 year follow-up. Another significant strength of the current study is the large sample size (n = 500), making our study the largest study to date investigating postoperative alignment after femur fracture fixation using the SIGN Fin nail.

Limitations of our study include the previously reported inconsistencies in quality and completeness of the SIGN database.^[6]^ However, since our primary outcome was radiographic alignment on immediate postoperative x-rays, we were able to ensure that all cases we included in the final cohort had adequate postoperative radiographs. In addition, there are certain limitations inherent to a retrospective study, such as recall bias. However, these are mitigated in part by the fact that the SOSD contains prospectively collected data. Finally, while our intra-rater reliability measurements showed moderate to good agreement (0.55 in the sagittal plane and 0.70 in the coronal plane), inter-rater reliability was much lower (0.32 in the sagittal plane and 0.37 in the coronal plane). Fortunately, the repeated measurements by the original reviewer identified a lower rate of malalignment >5° (10% vs 14%) with lower average DFAA measurements. Similarly, the separate measurements by the senior author identified an even lower rate of malalignment >5° (6%) and likewise lower average DFAA measurements. As such, the true rate of malalignment >5° in our cohort may actually have been lower than 9.4%.

Despite the number of projects that have evaluated the efficacy of the SIGN fin nail, numerous avenues remain open for future research. While we did not stratify results by hospital or institution, we did notice certain institutions had significantly higher volumes and accounted for up to 12.4% of all cases reviewed (62 of 500 cases). Comparisons between the higher and lower volume institutions could be used to evaluate the learning curve of the SIGN Fin nail with higher volume programs expected to achieve better results. Furthermore, we acknowledge that a variety of factors contribute to satisfactory (or unsatisfactory) postoperative alignment in long-bone fracture surgery. Regardless of the specific implant used, the experience of the surgeon and the surgical techniques utilized are critical factors that influence postoperative outcomes. Additionally, while Liu et al^[11]^ showed no significant differences in limb length discrepancy between fractures stabilized with the SIGN Fin nail and the standard SIGN nail at 1 year, studies are still needed to further evaluate postoperative shortening and rotational alignment both initially and with longer follow-up in patients with femur fractures stabilized with the SIGN Fin nail. In conclusion, our study adds to the growing body of literature supporting the utility of the SIGN Fin nail in achieving satisfactory postoperative alignment in femoral shaft fractures in both the coronal and sagittal planes on immediate postoperative radiographs.
